# Increased depression-related behavior during the postpartum period in inbred BALB/c and C57BL/6 strains

**DOI:** 10.1186/s13041-019-0490-z

**Published:** 2019-08-09

**Authors:** Hirotaka Shoji, Tsuyoshi Miyakawa

**Affiliations:** Division of Systems Medical Science, Institute for Comprehensive Medical Science, Fujita Health University, Toyoake, Aichi 470-1192 Japan

**Keywords:** Postpartum depression, Mouse model, Inbred substrain, Prepregnancy stress, BALB/cA, C57BL/6J

## Abstract

Pregnancy and lactation are characterized by dramatic changes in the endocrine system and brain in mammalian females. These changes, with stress before pregnancy, are potential risk factors for the development of postpartum depression (PPD). A valid animal model of PPD is needed to understand the neurobiological basis of the depressive state of females. To explore a mouse model of PPD, we first assessed anxiety-like and depression-related behaviors in nulliparous (virgin), nonlactating primiparous, and lactating primiparous females in four inbred strains of mice (C57BL/6J, C57BL/6JJcl, BALB/cAnNCrlCrlj, and BALB/cAJcl). Pups from the nonlactating female group were removed one day after parturition to examine the effects of physical interaction with pups on the postpartum behaviors. Second, we investigated the additional effects of prepregnancy stress (restraint stress for 6 h/day for 21 days) on postpartum behaviors in the BALB/cAJcl strain. We found that females of the two BALB/c substrains showed decreased locomotor activity and increased anxiety-like and depression-related behaviors compared with females of the two C57BL/6 substrains. Behavioral differences were also observed between the two substrains of each strain. Additionally, pregnancy- and lactation-dependent behavioral differences were found in some strains: lactating BALB/cAJcl females traveled shorter distance than the females of the other reproductive state groups, while nonlactating and lactating BALB/cAJcl and C57BL/6J females showed increased depression-related behavior compared with nulliparous females. Lactating BALB/cAJcl and C57BL/6JJcl females exhibited decreased sucrose preference or anhedonia-like behavior compared with nulliparous and nonlactating females, although these results did not reach statistical significance after correction for multiple testing. An additional independent experiment replicated the marked behavioral changes in lactating BALB/cAJcl females. Moreover, increased anxiety-like behavior was observed in lactating BALB/cAJcl females that experienced prepregnancy stress. These results suggest genetic contributions to the regulation of anxiety-like and depression-related behaviors in female mice. Furthermore, this study suggests that pregnancy and lactation cause decreased locomotor activity and increased depression-related behaviors, which was consistently found in our results, and that prepregnancy stress enhances anxiety-like behavior in the BALB/cAJcl strain. The inbred strain of female mice may be used as a potential model of PPD to further study the genetic and neurobiological mechanisms underlying the development of this disorder.

**Electronic supplementary material:**

The online version of this article (10.1186/s13041-019-0490-z) contains supplementary material, which is available to authorized users.

## Introduction

Pregnancy and lactation are characterized by significant changes in the endocrine system, brain, and behavior of females in mammals [1–3]. Becoming a mother is considered to be a significant experience accompanied by a positive mood, although this life event has the potential to be physically and psychologically exhausting and distressing and could possibly be a risk factor for developing postpartum depression (PPD) [4]. The prevalence of PPD is estimated to be approximately 10–15%, depending on the criteria used for diagnosis [5–7]. The symptoms of PPD include sadness, mood lability, irritability, restlessness, agitation, impaired concentration, and insomnia [8, 9]. Women with PPD often suffer from comorbid anxiety [9–11], and women with histories of prepartum depressive and anxiety symptoms are at increased risk for exacerbation or recurrence of the symptoms during the postpartum period [12–14]. Despite the high prevalence and negative outcomes, little is known regarding the etiology and pathophysiology of PPD; thus, a useful animal model needs to be developed for elucidating the risk factors and neurobiological mechanisms underlying PPD.

A number of studies have been conducted to identify genetic, neurobiological, and environmental factors contributing to depression mainly in male rodents [15, 16], although a few studies have focused on females during the postpartum period [1]. Various animal models of PPD have been created over the past two decades based on the theory of depression. Such models have mainly been developed in rats, including an ovarian-steroid-withdrawal model [17–19], a gestational stress model [20, 21], and a corticosterone-induced model [22, 23], with the exception of a recent report of a prepregnancy stress model in mice [24]. There are various inbred strains of mice that are genetically homogenous within each strain that have been used to study the contributions of genetic factors and their interactions with environmental factors in the brain and behavior. Among widely used inbred strains, C57BL/6 mice are considered to be more active and to be “less anxious”, whereas BALB/c mice are less active and are “more anxious” in novel environments, depending on the testing situations assessed for their behaviors [25–28]. C57BL/6 mice are also reported to be less susceptible to acute and chronic stress than BALB/c mice [29–35]. The two different strains of mice are considered to be a good animal model to assess genetic predisposition and environmental factors contributing to anxiety- and depression-like states in females.

The peripartum period is accompanied by dramatic changes in hormones and brain, which impacts various behaviors, including emotional behavior, in rodents [3, 4, 36, 37]. After parturition, interaction with pups is involved in the regulation of hormone release and emotional behavior [3, 4, 38]. Some studies have reported that in outbred strains of rats, lactating females exhibit less anxiety-like behavior than virgin females in different types of behavioral tests [39–42], whereas another report indicated that lactating female rats selectively bred for high and low anxiety-related behavior in the elevated plus maze test displayed higher anxiety-like behavior than virgin females in the hole-board test [38]. Similar to most previous findings in rats, lactating outbred Swiss female mice exhibited less anxiety-like behavior than nulliparous females in the light/dark transition test [43]. The reduced anxiety-like behavior during the postpartum period was suggested to be mediated by physical interactions with pups in rats [44]. However, the influences of interactions with pups during the lactation period with a depressive state or an anxiety-like state remain unknown in female mice of inbred C57BL/6 and BALB/c strains.

In this study, to investigate behavioral characteristics during the postpartum period in female mice, focusing on the genetic background and the effects of the mother-pup interaction, we first assessed anxiety-like and depression-related behaviors in nulliparous, nonlactating primiparous, and lactating primiparous females in four inbred strains of mice, including C57BL/6J, C57BL/6JJcl, BALB/cAnNCrlCrlj, and BALB/cAJcl mice. In the group of nonlactating females, pups were removed from the females one day after parturition to examine the influence of physical interactions with pups on the primiparous females. Their behaviors were analyzed in a battery of behavioral tests, including the light/dark transition test, the open field test, the elevated plus maze test, the Porsolt forced swim test, the tail suspension test, and the sucrose preference test, from postpartum days 3 to 10. Second, we investigated additional effects of prepregnancy stress on postpartum anxiety-like and depression-related behaviors in BALB/cAJcl females, which could be considered to be a potential mouse model of PPD, as indicated by the initial findings of this study.

## Materials and methods

### Animals

Naïve male and female mice of four strains (C57BL/6J, B6J; C57BL/6JJcl, B6JJcl; BALB/cAnNCrlCrlj, CACrlj; BALB/cAJcl, CAJcl) at 7 weeks of age were purchased from two vendors: Charles River Laboratories Japan and CLEA Japan, Inc. C57BL/6J and BALB/cAnNCrlCrlj mice were transported from the Hino Breeding Center of Charles River Laboratories Japan (Hino-cho, Gamo-gun, Shiga, Japan) to our animal facility, and C57BL/6JJcl and BALB/cAJcl mice were moved from the Fuji Breeding Center of the CLEA Japan, Inc. (Kamiyuno, Fujinomiya-shi, Shizuoka, Japan) to our animal facility. After arrival, mice were group-housed (two to four per cage) in plastic cages (250 × 182 × 139 mm) with paper chips for bedding (Paper Clean; Japan SLC, Inc., Shizuoka, Japan). Rooms were maintained under a 12-h light/dark cycle (lights on at 7:00 am) at 23 ± 2 °C. All animals were provided food (CRF-1, Oriental Yeast Co., Ltd., Tokyo, Japan) *ad libitum* throughout the experiments. All experimental procedures were approved by the Institutional Animal Care and Use Committee of Fujita Health University.

**Fig. 1:**
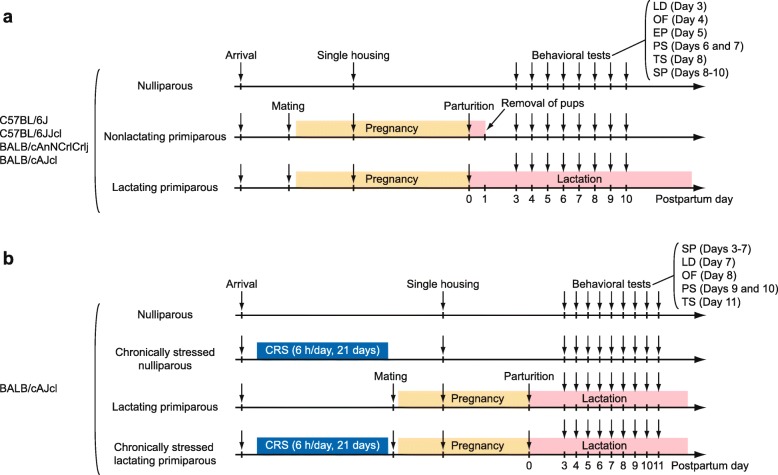
Schematic diagram of the experimental procedure. Flow diagrams of the experimental procedures for (**a**) behavioral comparisons of nulliparous, nonlactating primiparous, and lactating primiparous females of four inbred strains (C57BL/6J, C57BL/6JJcl, BALB/cAnNCrlCrlj, and BALB/cAJcl) of mice and for (**b**) behavioral analysis of nulliparous and lactating primiparous BALB/cAJcl females previously exposed to chronic restraint stress (CRS, for 6 h/day for 21 days). Behavioral analysis of female mice was performed using the light/dark transition (LD) test, the open field (OF) test, the elevated plus maze (EP) test, the Porsolt forced swim (PS) test, the tail suspension (TS) test, and the sucrose preference (SP) test

In the four strains, females were randomly divided into three groups: a nulliparous (virgin) group, a nonlactating primiparous group, and a lactating primiparous group (Fig. 1a). From 8 to 12 weeks of age, two females of the nulliparous group were group-housed in a cage. During the same time, two females in the nonlactating primiparous and lactating primiparous groups were mated with one male in a cage. Twelve days after mating, all females of the three groups were singly housed in plastic cages. The cages were checked every morning for the presence of pups, and the day of birth was designated as postpartum day 0 (PD0). On PD1, the pups were counted (mean litter size ± standard deviation; 6.23 ± 1.75 in B6J, 6.90 ± 1.22 in B6JJcl, 4.93 ± 1.27 in CACrlj, and 5.92 ± 1.70 in CAJcl), and pups in the nonlactating primiparous group were removed from their mothers. In each strain, 14–16 nulliparous, 11–14 nonlactating primiparous, and 11–17 lactating primiparous females were used for behavioral tests (for nulliparous, nonlactating primiparous, and lactating primiparous; *n* = 16, 13, and 17 in B6J; *n* = 14, 11, and 11 in B6JJcl; n = 16, 12, and 15 in CACrlj; n = 14, 14, and 13 in CAJcl). Females of the 4 strains were weighed on PD1, 3, and 8, and their food consumption (g) was measured from PD1 to PD3 and from PD8 to PD10 to analyze their energy intake during the postpartum period. The data pertaining to body weight and food consumption of the four females of each group in the CACrlj strain were excluded from the analysis because the data on PD1 were not recorded. From PD3 to PD10, behaviors were assessed with a battery of behavioral tests in the following order: the light/dark transition test (PD3), the open field test (PD4), the elevated plus maze test (PD5), the Porsolt forced swim test (PD6–7), the tail suspension test (PD8), and the sucrose preference test (PD8–10). To replicate the behavioral results for the BALB/cAJcl strain, an independent cohort of 12 nulliparous and 12 lactating primiparous females was included and subjected to the behavioral test battery in the same manner described above.

In another cohort of BALB/cAJcl mice, females were either left undisturbed (nonstressed group, NS) or exposed to restraint stress for 6 h/day for 21 consecutive days (chronic restraint stress group, CRS), starting at 10 weeks of age (Fig. 1b). Mice were restrained by placing them in a well-ventilated 50 mL polypropylene conical tube (114.4 mm long, 29.1 mm outer diameter; Corning Inc., NY, USA) with a quarter of a paper towel (Kim Towel, Nippon Paper Crecia Co., Ltd., Tokyo, Japan) placed in the tube to fill a space between the mouse and the tube cap. On the following day after chronic stress, approximately half of the female groups were mated with naïve males. Nine days after mating, all females were singly housed. After parturition, the four groups, i.e., NS-nulliparous, CRS-nulliparous, NS-lactating, and CRS-lactating females (*n* = 14, 14, 13, and 9, respectively), were subjected to a battery of behavioral tests in the following order: the sucrose preference test (PD3–7), the light/dark transition test (PD7), the open field test (PD8), the Porsolt forced swim test (PD9–10), and the tail suspension test (PD11). Females were weighed weekly during the stress period, and their body weights (g) and food consumption (g) were monitored daily after they were singly housed. The body weight and food consumption data during the peripartum period (PD-7, PD-1, PD1, PD7, and PD14) were analyzed.

In the three different cohorts of mice used in this study, females belonging to any of the reproductive states (nulliparous, nonlactating primiparous, and lactating primiparous groups) were not subjected to behavioral tests until the beginning of the behavioral test battery that was performed during the postpartum period (Fig. 1).

### Behavioral test battery

Female mice in each cohort were subjected to a battery of behavioral tests in the order described above. After each test, the floors and the walls of the testing apparatuses were cleaned with hypochlorous acid water to prevent bias based on olfactory cues. The behavioral tests were performed between 9:00 am and 1:00 pm. In the tail suspension test, the behavioral data of one nulliparous B6J female and one nonlactating primiparous B6J female were lost due to a technical problem related to the video camera settings of the image analysis system.

### Light/dark transition test

The light/dark transition test, developed by Crawley and colleagues [45], was performed as previously described [46]. The apparatus consisted of a cage (21 × 42 × 25 cm) divided into two sections of equal size by a partition with a door (O’Hara & Co., Tokyo, Japan). One chamber was with white plastic walls and was brightly illuminated (390 lx) by lights attached above the ceiling of the chamber. The other chamber was with black plastic walls and was dark (2 lx). The two chambers had a gray plastic floor. Mice were placed into the dark chamber and were allowed to move freely between the two chambers for 10 min with the door open. The distance traveled (cm), total number of transitions, latency to first enter the light chamber (s), and time spent in the light chamber (s) were recorded automatically using the ImageLD program (see “Image analysis”).

### Open field test

The open field test [47] was conducted in the open field apparatus (40 × 40 × 30 cm; Accuscan Instruments, Columbus, OH, USA), in which the floor of center area was illuminated at 100 lx. The center area was defined as a 20 cm × 20 cm area. Each mouse was placed in one corner of an open field. The total distance traveled (cm), vertical activity (rearing measured by counting the number of photobeam interruptions), time spent in the center area (s), and stereotypic counts (beam-break counts for stereotyped behaviors) were recorded for the total 30-min period, and analyzed in each 10-min block.

### Elevated plus maze test

The elevated plus maze test, which has been widely used to assess anxiety [48], was performed as previously described [49]. The apparatus consisted of two open arms (25 × 5 cm) and two enclosed arms of the same size with 15-cm-high transparent walls and a central square (5 × 5 cm) connecting the arms (O’Hara & Co., Tokyo, Japan). The floors of arms and central square were made of gray plastic plates and were elevated to a height of 55 cm above the floor. To prevent mice from falling off the open arms, those arms were surrounded by a raised ledge (3-mm thick and 3-mm high). Arms of the same type were located opposite one another. Each mouse was placed in the central square of the maze facing one of the closed arms. The number of arm entries, distance traveled (cm), percentage of entries into open arms, and percentage of time spent in open arms were measured during a 10-min test period. Data acquisition and analysis were performed automatically using the ImageEP program.

### Porsolt forced swim test

The Porsolt forced swim test, developed by Porsolt and colleagues [50], was performed to assess depression-related behavior. Mice were placed into a Plexiglas cylinder (20 cm height × 10 cm diameter, O’Hara & Co., Tokyo, Japan) filled with water (approximately 21 °C) up to a height of 7.5 cm for 10 min per day for consecutive 2 days. The percentage of immobility time was recorded automatically using the ImagePS program.

### Tail suspension test

The tail suspension test was used to evaluate depression-related behavior [51]. Mice were suspended 30 cm above the floor in a visually isolated area by adhesive tape placed approximately 1 cm from the tip of the tail. Immobility was recorded for a 10-min test period. Immobility time was measured automatically using the ImageTS program. The immobility times in B6 substrains were not analyzed due to the large number of mice displaying tail-climbing behavior as described in the Results section. For the two BALB/c substrains, a statistical analysis of immobility time was performed after excluding the data of mice showing tail-climbing behavior more than 30 s (an arbitrary threshold).

### Sucrose preference test

In the sucrose preference test for four strains, mice were given a bottle containing water and a second with 1% sucrose solution with the left/right location balanced across groups of animals in their home cages after the tail suspension test on PD8. Bottles were weighed prior to testing and again approximately 48 h after the start of the test (PD8–10). In another cohort of BALB/cAJcl females with or without stress experience, the sucrose preference test was conducted by the slightly modified version of the procedure: mice were provided with two bottles containing either water or 2% sucrose for successive four days with the left-right positions of the bottles alternated daily (PD3–7). Sucrose preference was expressed as 100 × [(sucrose intake)/(sucrose intake + water intake)].

### Image analysis

For some behavioral tests, image analysis programs (ImageLD/EP/PS/TS) were used to automatically analyze mouse behaviors. The application programs, based on the public domain ImageJ program (developed by Wayne Rasband at the National Institute of Mental Health, Bethesda), were developed and modified for each test by Tsuyoshi Miyakawa. The ImageLD/EP programs can be freely downloaded from the “Mouse Phenotype Database” (http://www.mouse-phenotype.org/).

### Statistical analysis

Statistical analyses were performed using SAS University Edition (SAS Institute, Cary, NC, USA). The data of four strains were analyzed using a two-way ANOVA with strain (B6J, B6JJcl, CACrlj, CAJcl) and reproductive state (nulliparous, non-lactating primiparous, and lactating primiparous) as between subject variables or a three-way repeated-measures ANOVA with Strain and Reproductive state as between subject variables and with Time as a within subject variable. The data of BALB/cAJcl females with or without stress experience were analyzed with two- or three-way ANOVAs with factors of Reproductive state (nulliparous, lactating primiparous), Stress experience (no stress, chronic restraint stress), and Time. We defined “study-wide significance” as statistical significance that survived the Benjamini-Hochberg False Discovery Rate (FDR) correction [52, 53] for controlling for multiple hypothesis testing based on the number of behavioral measures of the test battery. “Nominal significance” was defined as having achieved statistical significance without the FDR correction (uncorrected *p* < 0.05). When significant interactions between two or three factors were observed, simple interactions and simple main effects were examined as appropriate. Post-hoc multiple comparisons were further performed using Fisher’s LSD with Bonferroni correction for multiple comparison. Values in graphs are expressed as the mean ± SEM.

## Results

### Locomotor activity and anxiety-like behavior of BALB/c and C57BL/6 female mice during the postpartum period in the open field test

**Fig. 2:**
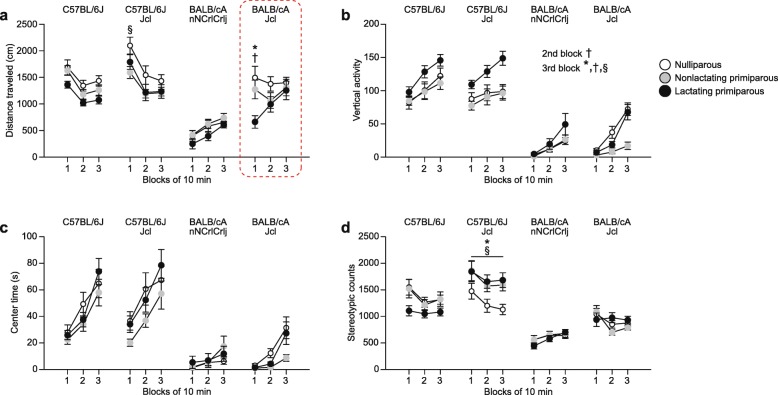
Locomotor activity and anxiety-like behavior of BALB/c and C57BL/6 female mice during the postpartum period in the open field test. The open field test was performed for a 30-min test period in nulliparous, nonlactating primiparous, and lactating primiparous females of C57BL/6J, C57BL/6JJcl, BALB/cAnNCrlCrlj, and BALB/cAJcl strains. **a**–**d** Open field test: **a** distance traveled (cm), **b** vertical activity, **c** center time (s), and **d** stereotypic counts during three 10-min blocks. Values are means ± SEM. The dashed enclosure in the figure indicates the results replicated in another independent experiment (see Fig. 7). In each strain, *p < 0.05 after Bonferroni correction (lactating primiparous vs.  nulliparous); †*p* < 0.05 after Bonferroni correction (lactating primiparous vs. nonlactating primiparous); §*p* < 0.05 after Bonferroni correction (nonlactating vs. nulliparous)

The statistical results of each behavioral test are summarized in Additional file 1: (Table S1). Three-way repeated measures ANOVA showed that there were significant main effects of strain (*F*_3,154_ = 48.73, *p* < 0.0001) and reproductive state (*F*_2,154_ = 8.42, *p* = 0.0003), significant interactions of strain × time (*F*_6,308_ = 27.60, *p* < 0.0001) and reproductive state × time (*F*_4,308_ = 2.80, *p* = 0.0262), and a significant strain × reproductive state × time interaction (*F*_12,308_ = 2.39, *p* = 0.0059) on distance traveled (Fig. 2a). In each 10-min block, significant strain differences were found in the respective reproductive state groups; females of the two B6 substrains traveled significantly longer distances than CACrlj females in each reproductive state group (all *p* < 0.0083), although the difference between nonlactating B6JJcl and CACrlj females in the third 10-min block did not reach significance after Bonferroni correction (*p* = 0.0106). In addition, in the first 10-min block, nonlactating and lactating B6J females traveled longer distances than nonlactating and lactating CAJcl females (*p* = 0.0373 and *p* < 0.0001, respectively), and nulliparous and lactating B6JJcl females traveled longer distances than nulliparous and lactating CAJcl females (*p* = 0.0003 and *p* < 0.0001, respectively). Significant strain differences were also found in the distance traveled between BALB/c substrains; CAJcl females traveled longer distances than CACrlj females in each reproductive state group and in each 10-min block (all *p* < 0.0083, except for the following cases: the lactating group in the first 10-min block, *p* = 0.0141; the nonlactating group in the second 10-min block, *p* = 0.0120). For B6 substrains, longer distances traveled were observed in nulliparous and lactating B6JJcl females than in nulliparous and lactating B6J females, although the differences did not reach significance after correction for multiple comparisons (for nulliparous, *p* = 0.0119; for lactating, *p* = 0.0130). Furthermore, in the first 10-min block, a significant difference was found between reproductive state groups in B6JJcl mice (nonlactating < nulliparous, *p* = 0.0048) and in CAJcl mice (lactating < nulliparous and nonlactating, *p* < 0.0001 and *p* = 0.0004, respectively). The decreased distance traveled in lactating CAJcl females and other behavioral results were replicated in another independent cohort of mice, as described in detail below (see section “Replication of behavioral results in lactating BALB/cAJcl mice”; the dashed enclosures in Fig. 2a and other figures indicate the replicated results in CAJcl females).

For vertical activity (Fig. 2b), there were significant main effects of strain (*F*_3,154_ = 130.74, *p* < 0.0001) and reproductive state (*F*_2,154_ = 11.01, *p* < 0.0001), a significant strain × time interaction (*F*_6,308_ = 2.99, *p* = 0.0074), and a significant reproductive state × time interaction (*F*_4,308_ = 5.91, *p* = 0.0001). In each 10-min block, the two B6 substrains showed more vertical activity than the two BALB/c substrains (all *p* < 0.0001). There was a significant difference between BALB/c substrains in the third 10-min block; CAJcl females exhibited more vertical activity than CACrlj females (*p* = 0.0071). In the second and third 10-min blocks, there were significant differences in vertical activity among females of different reproductive state groups. Lactating females showed more vertical activity than nulliparous and nonlactating females (for the second block, *p* = 0.0428 and *p* = 0.0007, respectively; for the third block, *p* = 0.0001 and *p* < 0.0001, respectively), and nulliparous females showed more vertical activity than nonlactating females in the third 10-min block (*p* = 0.0077).

Regarding center time (Fig. 2c), there was a significant main effect of strain (*F*_3,154_ = 47.17, *p* < 0.0001) and a significant strain × time interaction (*F*_6,308_ = 9.34, *p* < 0.0001). In each 10-min block, the two B6 substrains spent more time in the center area than the two BALB/c substrains (all *p* < 0.0001). In the third 10-min block, CAJcl females spent more time in the center area than CACrlj females, although the difference did not reach study-wide significance (*p* = 0.0470). There were no significant differences in center time between B6 substrains or among females of different reproductive states.

There was a significant main effect of strain (*F*_3,154_ = 62.00, *p* < 0.0001) and a significant strain × reproductive state interaction (*F*_6,154_ = 3.70, *p* = 0.0018) on stereotypic counts (Fig. 2d). The two B6 substrains showed more stereotypic counts than the two BALB/c substrains in each reproductive state (all *p* < 0.0083), except for the case of the comparison between lactating B6J and lactating CAJcl females (*p* = 0.2843). There were also significant differences in stereotypic counts between B6 substrains and between BALB/c substrains, with more stereotypic counts in B6JJcl females than in B6J females in the nonlactating and lactating groups (for nulliparous*, p* = 0.0370; for nonlactating*, p* = 0.0194; for lactating, *p* < 0.0001) and with more stereotypic counts in CAJcl females than in CACrlj females in the nulliparous, nonlactating, and lactating groups (*p* = 0.0121, *p* = 0.0833, and *p* = 0.0036, respectively). Significant differences in stereotypic counts were found among groups of different reproductive states in the B6JJcl strain. In the B6JJcl strain, nonlactating and lactating females showed more stereotypic counts than nulliparous females (*p* = 0.0027 and *p* = 0.0007, respectively).

### Locomotor activity and anxiety-like behavior of BALB/c and C57BL/6 female mice during the postpartum period in the light/dark transition test

**Fig. 3:**
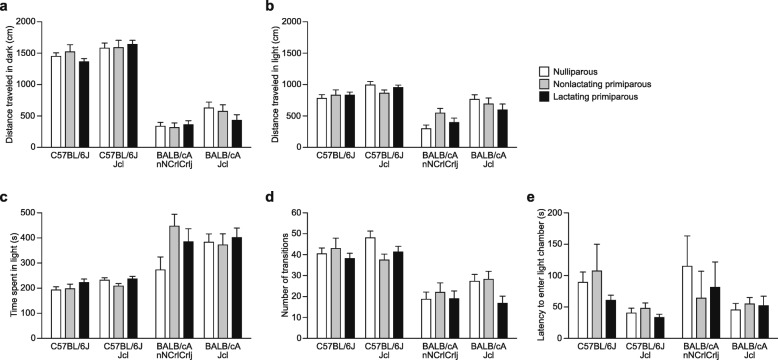
Locomotor activity and anxiety-like behavior of BALB/c and C57BL/6 female mice during the postpartum period in the light/dark transition test. The light/dark transition test was performed for a 10-min test period in nulliparous, nonlactating primiparous, and lactating primiparous females of C57BL/6J, C57BL/6JJcl, BALB/cAnNCrlCrlj, and BALB/cAJcl strains. **a**–**d** Light/dark transition test: **a** distance traveled (cm) in the dark chamber, **b** distance traveled (cm) in the light chamber, **c** time spent in the light chamber (s), **d** number of transitions between the light and dark chambers, and **e** latency to enter the light chamber (s). Values are means ± SEM

In the light/dark transition test, there were significant main effects of strain in the distance traveled in the dark and light boxes (Fig. 3a: for the dark box, *F*_3,154_ = 182.62, *p* < 0.0001; Fig. 3b: for the light box, *F*_3,154_ = 30.33, *p* < 0.0001), in the time spent in the light box (Fig. 3c: *F*_3,154_ = 23.10, *p* < 0.0001), and in the number of transitions between boxes (Fig. 3d: *F*_3,154_ = 31.16, *p* < 0.0001). No significant effect of strain was found in the latency to enter the light box (Fig. 3e: *F*_3,154_ = 2.17, *p* = 0.0944). Post hoc strain comparisons revealed that the two B6 substrains traveled longer distances in each box, spent shorter amounts of time in the light box, and had more transitions than the two BALB/c substrains, although the difference in the distance traveled in the light box between the B6J and CAJcl strains did not reach study-wide significance (*p* < 0.0083). In addition, B6JJcl mice traveled longer distances than B6J mice in the dark and light boxes (*p* = 0.0198 and *p* = 0.0364, respectively; the differences did not reach study-wide significance), and CAJcl mice traveled longer distances than CACrlj mice in the dark and light boxes (*p* = 0.0019 and *p* < 0.0001, respectively). There were no significant main effects of reproductive state and no significant strain × reproductive state interaction in any of the behavioral measures (see Additional file 1: Table S1), indicating that there were no behavioral differences in the light/dark transition test among females of different reproductive state groups in each strain.

### Locomotor activity and anxiety-like behavior of BALB/c and C57BL/6 female mice during the postpartum period in the elevated plus maze test

**Fig. 4:**
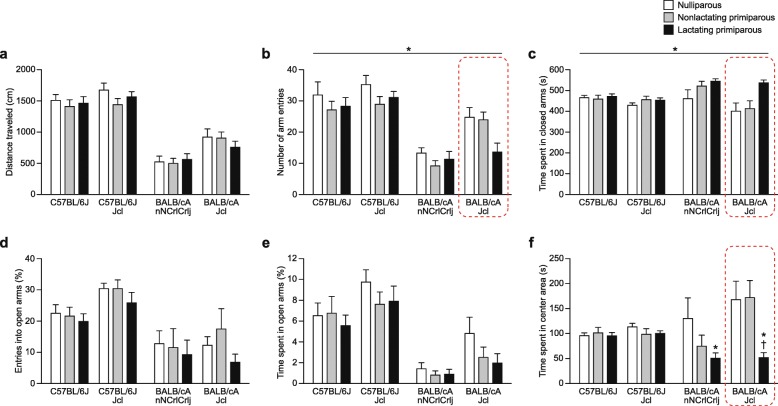
Locomotor activity and anxiety-like behavior of BALB/c and C57BL/6 female mice during the postpartum period in the elevated plus maze test. The elevated plus maze test was performed for a 10-min test period in nulliparous, nonlactating primiparous, and lactating primiparous females of C57BL/6J, C57BL/6JJcl, BALB/cAnNCrlCrlj, and BALB/cAJcl strains. **a**–**f** Elevated plus maze test: **a** distance traveled (cm), **b** number of total arm entries, **c** time spent in closed arms (s), **d** entries into open arms (%), **e** time spent in open arms (%), and **f** time spent in center area (s). Values are means ± SEM. Dashed enclosures in the figure indicate the results replicated in another independent experiment (see Fig. 7). *p < 0.05 after Bonferroni correction (lactating primiparous vs. nulliparous); †*p* < 0.05 after Bonferroni correction (lactating primiparous vs. nonlactating primiparous)

There were significant main effects of strain in distance traveled (Fig. 4a; *F*_3,154_ = 68.93, *p* < 0.0001), total number of arm entries (Fig. 4b; *F*_3,154_ = 32.15, *p* < 0.0001), percentage of open arm entries (Fig. 4d; F_3,154_ = 13.38, *p* < 0.0001), time spent in closed arms (Fig. 4c; strain, *F*_3,154_ = 3.94, *p* = 0.0096), and percentage of time spent in open arms (Fig. 4e; *F*_3,154_ = 25.26, *p* < 0.0001), while there was no significant main effect of strain in time spent in the center area (*F*_3,154_ = 2.26, *p* = 0.0841). Post hoc strain comparisons revealed that the two B6 substrains showed longer distance traveled, a greater number of total arm entries, a higher percentage of open arm entries, and a higher percentage of time spent in open arms than the two BALB/c substrains (all *p* < 0.0083), and the two B6 substrains, B6J and B6JJcl, spent less time in closed arms than the CACrlj substrain (*p* = 0.0288 and *p* = 0.0032, respectively). In addition, there was a tendency toward differences between the C57BL/6 substrains, with higher percentages of open arm entries and of open arm time in the B6JJcl substrain than in the B6J substrain (*p* = 0.0211 and *p* = 0.0187, respectively). In BALB/c mice, significant substrain differences were found for almost all of the measures, with longer distance traveled, a greater number of arm entries, less time spent in closed arms, and more time spent in open arms in CAJcl mice than in CACrlj mice (*p* < 0.0001, *p* < 0.0001, *p* = 0.0044, and *p* = 0.0235, respectively).

Significant main effects of reproductive state were found in the number of total arm entries (*F*_2,154_ = 3.93, *p* = 0.0216) and the time spent in closed arms (*F*_2,154_ = 6.67, *p* = 0.0017). Post hoc comparisons revealed fewer total arm entries by nonlactating and lactating primiparous females than nulliparous females (*p* = 0.0477 and *p* = 0.0083, respectively), although the difference between nulliparous and nonlactating females did not reach study-wide significance. Post hoc analyses also revealed that lactating females spent more time in closed arms than nulliparous females (*p* = 0.0004). The time spent in closed arms was also higher in lactating females than in nonlactating females (*p* = 0.0316), which may be partially dependent on strain, especially the CAJcl substrain, as indicated by a marginally significant strain × reproductive state interaction (*F*_6,154_ = 2.06, *p* = 0.0607).

For the time spent in the center area, a significant strain × reproductive state interaction was found (strain effect, *F*_3,154_ = 2.26, *p* = 0.0841; reproductive state effect, *F*_2,154_ = 6.02, *p* = 0.0030; strain × reproductive state interaction, *F*_6,154_ = 2.39, *p* = 0.0307). In the two BALB/c substrains, lactating females spent less time in the center area than nulliparous females (for CACrlj, *p* = 0.0078; for CAJcl, *p* = 0.0003). In the CAJcl substrain, lactating females also spent less time in the center area than nonlactating females (*p* = 0.0002).

### Depression-related behavior in BALB/c and C57BL/6 female mice during the postpartum period in the forced swim test

**Fig. 5:**
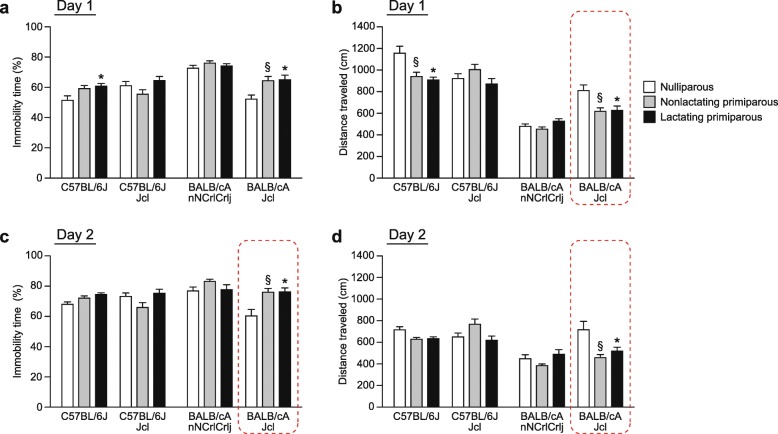
Depression-related behavior in BALB/c and C57BL/6 female mice during the postpartum period in the forced swim test. The forced swim test was performed for a 10-min test period in nulliparous, nonlactating primiparous, and lactating primiparous females of C57BL/6J, C57BL/6JJcl, BALB/cAnNCrlCrlj, and BALB/cAJcl strains. **a**–**d** Porsolt forced swim test: **a** immobility time (%) on day 1, **b** distance traveled (cm) on day 1, **c** immobility time (%) on day 2, and **d** distance traveled (cm) on day 2. Values are means ± SEM. Dashed enclosures in the figure indicate the results replicated in another independent experiment (see Fig. 7). In each strain, *p < 0.05 after Bonferroni correction (lactating primiparous vs. nulliparous); §*p* < 0.05 after Bonferroni correction (nonlactating primiparous vs. nulliparous)

Three-way repeated measures ANOVAs revealed significant main effects of strain and reproductive state and significant strain × reproductive state interactions in the percentage of immobility time and distance traveled on test days 1 and 2, while no significant strain × reproductive state × time interactions were observed for any of the behavioral measures (for details of the statistical results, see Additional file 1: Table S1). On test day 1, in each reproductive state group, there were significant strain effects on the percentage of immobility time (Fig. 5a and Additional file 2: Figure S1A) and distance traveled (Fig. 5b and Additional file 2: Figure S1B). Post hoc strain comparison revealed that CACrlj females showed higher immobility and shorter distance traveled than females of B6 substrains irrespective of their reproductive states (for immobility, CACrlj > B6J and B6JJcl, all *p* < 0.0083, except for the comparison between lactating B6JJcl females and lactating CACrlj females, for which the *p* value was 0.0088; for distance traveled, CACrlj < B6J and B6JJcl, all *p* < 0.0001). Nulliparous CAJcl females showed lower immobility than nulliparous B6JJcl females (*p* = 0.0107), while nonlactating CAJcl females showed higher immobility than nonlactating B6JJcl females (*p* = 0.0156), although the differences did not reach study-wide significance. In addition, CAJcl females traveled shorter distances than B6J females (for nulliparous, *p* < 0.0001; for nonlactating, *p* < 0.0001; for lactating, *p* < 0.0001) and B6JJcl females (for nulliparous, *p* = 0.0551; for nonlactating, *p* < 0.0001; for lactating, *p* = 0.0001). Post hoc substrain comparison indicated that there were substrain differences in immobility and distance traveled. Higher immobility and shorter distances traveled were observed in B6JJcl mice than in B6J mice only in the nulliparous group (for immobility, *p* = 0.0042; for distance traveled, *p* < 0.0001). For BALB/c substrains, higher immobility and shorter distances traveled were observed in CACrlj females than in CAJcl females in the nulliparous group (for immobility, *p* < 0.0001; for distance traveled, *p* < 0.0001), the nonlactating group (for immobility, *p* = 0.0014; for distance traveled, *p* = 0.0067), and the lactating group (for immobility, *p* = 0.0091; for distance traveled, *p* = 0.0881). In the B6J and CAJcl groups, there were significant effects of reproductive state on the percentage of immobility time and distance traveled; nonlactating and lactating females exhibited higher immobility and shorter distances traveled than nulliparous females in the B6J group (for immobility, nonlactating and lactating > nulliparous, *p* = 0.0234 and *p* = 0.0034; for distance traveled, nonlactating and lactating < nulliparous, *p* = 0.0002 and *p* < 0.0001) and in the CAJcl group (for immobility, nonlactating and lactating > nulliparous, *p* = 0.0005 and *p* = 0.0004; for distance traveled, nonlactating and lactating < nulliparous, *p* = 0.0011 and *p* = 0.0020).

On test day 2, there were significant main effects of strain and reproductive state and a significant strain × reproductive state interaction on the percentage of immobility time and distance traveled (for details of the statistical results, see Additional file 1: Table S1). In the nulliparous group, CACrlj females showed more immobility and shorter distances traveled than B6J and B6JJcl females (for immobility, CACrlj > B6J, *p* = 0.0092; for distance traveled, CACrlj < B6J and B6JJcl, *p* < 0.0001 and *p* = 0.0002), and CAJcl females exhibited less immobility than B6J and/or B6JJcl females (CAJcl < B6J and B6JJcl, *p* = 0.0295 and *p* = 0.0004). Nulliparous CACrlj females showed more immobility and shorter distance traveled than nulliparous CAJcl females (all *p* < 0.0001). Similar differences between CACrlj and B6 substrains were found in the nonlactating group for immobility (CACrlj > B6J and B6JJcl, *p* = 0.0042 and *p* < 0.0001) and for distance traveled (CACrlj < B6J and B6JJcl, all *p* < 0.0001). In contrast to the nulliparous group, nonlactating CAJcl females displayed more immobility and shorter distances traveled than B6 substrains (for immobility, CAJcl > B6JJcl, *p* = 0.0092; for distance traveled, CAJcl < B6J and B6JJcl, *p* = 0.0023 and *p* < 0.0001). In addition, nonlactating B6J females showed shorter distance traveled than nonlactating B6JJcl females (*p* = 0.0191). In the lactating group, CACrlj and CAJcl females showed shorter distances traveled than either B6 substrains or both (CACrlj < B6J and B6JJcl, *p* = 0.0051 and *p* = 0.0244; CAJcl < B6J, *p* = 0.0311). The post hoc comparisons further indicated that there were significant differences among females of different reproductive state groups. In the B6JJcl group, lactating females showed more immobility than nonlactating females (*p* = 0.0205), and nulliparous and lactating females traveled shorter distances than nonlactating females (*p* = 0.0423 and *p* = 0.0159, respectively), although the differences did not reach a significance after Bonferroni correction. In the CAJcl group, nonlactating and lactating females showed more immobility and shorter distances traveled than nulliparous females (for immobility, all *p* < 0.0001; for distance traveled, *p* = 0.0005 and *p* < 0.0001).

### Depression-related behavior in BALB/c and C57BL/6 female mice during the postpartum period in the tail suspension test

In the tail suspension test, tail-climbing behavior was frequently observed in females of C57BL/6 substrains, as previously reported in male C57BL6J mice [54]. The total time of tail-climbing behavior was measured by an experimenter blinded to the reproductive state using video images recorded during the testing period. The number of mice showing tail-climbing behavior for more than 30 s (an arbitrary threshold) in total during the testing period were counted. Most B6J and B6JJcl females showed tail-climbing behavior for more than 30 s, while fewer BALB/c females displayed behavior over the threshold time (Additional file 1: Table S2). Two-way repeated measures ANOVA showed that there were significant main effects of strain (*F*_3,152_ = 22.16, *p* < 0.0001) and reproductive state (*F*_2,152_ = 8.62, *p* = 0.0003) but no significant strain × reproductive state interaction (*F*_6,152_ = 2.09, *p* = 0.0573) on the percentage of time of tail-climbing behavior (Additional file 3: Figure S2A). Post hoc strain comparisons revealed that B6 females showed higher percentages of tail-climbing behavior than BALB/c females (B6J and B6JJcl > CACrlj and CAJcl, all *p* < 0.0001). There were no significant differences in the percentage of tail-climbing behavior between B6 substrains or between BALB/c substrains (B6J vs. B6JJcl, *p* = 0.4733; CACrlj vs. CAJcl, *p* = 0.3485). The immobility times in B6 substrains were not analyzed due to the large number of mice displaying tail-climbing behavior. For the two BALB/c substrains, a statistical analysis of immobility time was performed after excluding the data of mice showing tail-climbing behavior more than 30 s. The analysis revealed that there was a significant main effect of strain on the percentage of immobility time (*F*_1,66_ = 5.62, *p* = 0.0206), while there was no significant main effect of reproductive state (*F*_2,66_, = 1.60, *p* = 0.2105) and no significant strain × reproductive state interaction (*F*_2,66_ = 0.08, *p* = 0.9204). Post hoc comparison revealed that CACrlj females exhibited more immobility than CAJcl females (Additional file 3: Figure S2B).

### Anhedonia-like behavior in BALB/c and C57BL/6 female mice during the postpartum period in the sucrose preference test

**Fig. 6:**
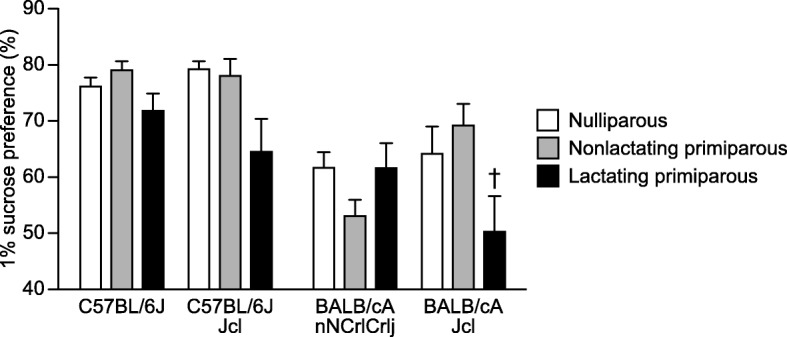
Anhedonia-like behavior in BALB/c and C57BL/6 female mice during the postpartum period in the sucrose preference test. The sucrose preference test was performed in nulliparous, nonlactating primiparous, and lactating primiparous females of C57BL/6 J, C57BL/6 JJcl, BALB/cAnNCrlCrlj, BALB/cAJcl strains. Water intake and 1% sucrose intake were measured for two days, and sucrose preference (%) was calculated according to the formula: 100 × (sucrose intake/total solution intake). Values are means ± SEM. †*p* < 0.05 after Bonferroni correction (lactating primiparous vs. nonlactating primiparous)

Two-way ANOVA showed that there were significant main effects of strain (*F*_3,154_ = 16.37, *p* < 0.0001) and reproductive state (*F*_2,154_ = 6.21, *p* = 0.0025) and a significant strain × reproductive state interaction (*F*_6,154_ = 2.67, *p* = 0.0172) in the percentage of sucrose preference (Fig. 6). In most cases, when the two B6 substrains were compared with the BALB/c substrains, B6 females showed a higher percentage of sucrose preference than BALB/c females in the nulliparous group (B6J > CACrlj and CAJcl, *p* = 0.0035 and *p* = 0.0188; B6JJcl > CACrlj and CAJcl, *p* = 0.0007 and *p* = 0.0043), the nonlactating group (B6J > CACrlj and CAJcl, *p* < 0.0001 and *p* = 0.0646; B6JJcl > CACrlj and CAJcl, *p* < 0.0001 and *p* = 0.1140), and the lactating group (B6J > CACrlj and CAJcl, *p* = 0.0376 and *p* < 0.0001; B6JJcl > CACrlj and CAJcl, *p* = 0.5972 and *p* = 0.0128). There were no significant differences in sucrose preference between the B6 substrains and the BALB/c substrains irrespective of their reproductive states, except for nonlactating CACrlj females, which showed lower sucrose preference than nonlactating CAJcl females (*p* = 0.0035). Significant differences in sucrose preference among reproductive state groups were found in the B6JJcl and CAJcl groups. In the B6JJcl group, lactating females showed a lower percentage of sucrose preference than nulliparous and nonlactating females, although the differences failed to reach significance after Bonferroni correction (*p* = 0.0088 and *p* = 0.0228, respectively). In the CAJcl group, lactating females exhibited a significantly lower percentage of sucrose preference than nonlactating females (*p* = 0.0005) and showed a marginally significant decrease in the percentage of sucrose preference compared with nulliparous females (*p* = 0.0098).

### Replication of behavioral results in lactating BALB/cAJcl mice

**Fig. 7:**
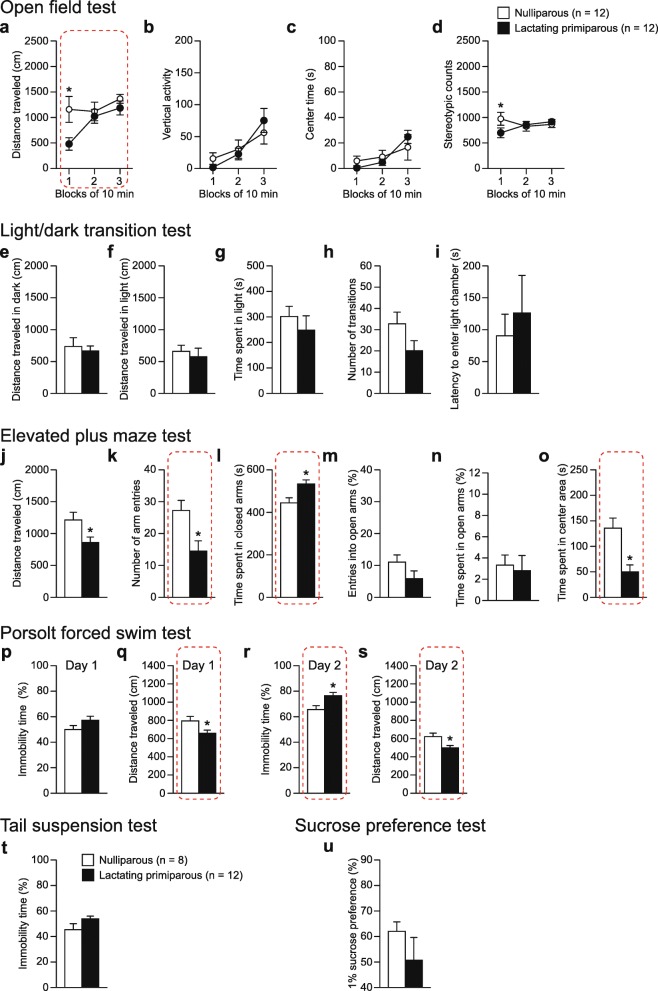
Replication of behavioral results in BALB/cAJcl female mice. A series of behavioral tests was performed in independent groups of nulliparous and lactating primiparous BALB/cAJcl females (*n* = 12, each group). **a**–**d** Open field test: **a** distance traveled (cm), **b** vertical activity, **c** center time (s), and **d** stereotypic counts during three 10-min blocks. **e**–**i** Light/dark transition test: **e** distance traveled (cm) in the dark chamber, **f** distance traveled (cm) in the light chamber, **g** time spent in the light chamber (s), **h** number of transitions between the light and dark chambers, and **i** latency to enter the light chamber (s). **j**–**o** Elevated plus maze test: **j** distance traveled (cm), **k** number of total arm entries, **l** time spent in closed arms (s), **m** entries into open arms (%), **n** time spent in open arms (%), and **o** time spent in center area (s). **p**–**s** Porsolt forced swim test: **p** immobility time (%) on day 1, **q** distance traveled (cm) on day 1, **r** immobility time (%) on day 2, and **s** distance traveled (cm) on day 2. **t** Immobility time (%) in the tail suspension test. Females showing tail-climbing behavior for more than 30 s in total during the test period were excluded from the analysis. **u** 1% sucrose preference (%). Values are means ± SEM. Dashed enclosures in the figure indicate the results replicated in another independent experiment (see Figs. 2, 4, and 5). *p < 0.05, compared with nulliparous females

The series of behavioral tests revealed multiple behavioral changes after parturition, including decreased locomotor activity in the open field and elevated plus maze tests and increased depression-related behavior in the forced swim test, in BALB/cAJcl female mice. These findings were replicated in an additional independent experiment in which behavioral comparisons were made between the two groups of 12 nulliparous and 12 lactating BALB/cAJcl females (Fig. 7), although the statistical results in almost all behavioral measures did not reach a study-wide significance level after FDR correction (for the detailed statistical results, see Additional file 1: Table S3). Although lactating females showed lower sucrose preference than nulliparous females, the result failed to reach statistical significance in this experiment. The two independent experiments of this study strengthen the validity of the findings of decreased locomotor activity and increased depression-related behavior in BALB/cAJcl lactating females.

### Increased anxiety-like behavior in lactating BALB/cAJcl mice that experienced prepregnancy stress

**Fig. 8:**
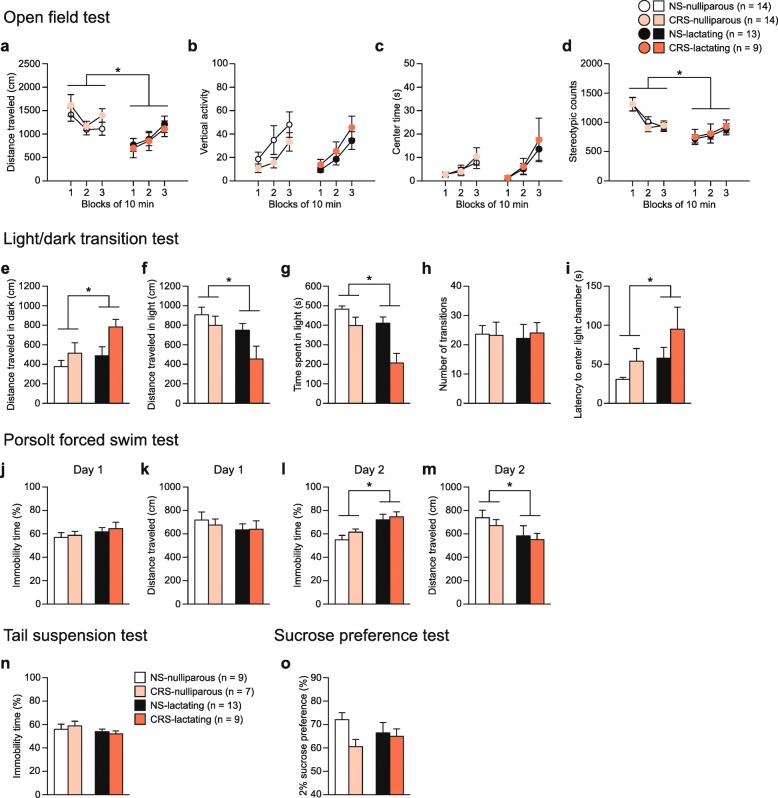
Increased anxiety-like behavior in BALB/cAJcl female mice previously exposed to chronic restraint stress. Behaviors of nulliparous and lactating primiparous BALB/cAJcl females previously exposed to chronic restraint stress were assessed (nonstressed (NS) nulliparous females, *n* = 14; chronic restraint-stressed (CRS) nulliparous females, n = 14; NS-lactating females, *n* = 13; CRS-lactating females, *n* = 9). **a**–**d** Open field test: **a** distance traveled (cm), **b** vertical activity, **c** center time (s), and **d** stereotypic counts during three 10-min blocks. **e**–**i** Light/dark transition test: **e** distance traveled (cm) in the dark chamber, **f** distance traveled (cm) in the light chamber, **g** time spent in the light chamber (s), **h** number of transitions between the light and dark chambers, and **i** latency to enter the light chamber (s). **j**–**m**) Porsolt forced swim test: **j** immobility time (%) on day 1, **k** distance traveled (cm) on day 1, **l** immobility time (%) on day 2, and **m** distance traveled (cm) on day 2. **n** Immobility time (%) in the tail suspension test. Females showing tail-climbing behavior for more than 30 s in total during the test period were excluded from the analysis. **o** 2% sucrose preference (%). Values are means ± SEM. *p < 0.05, compared with nulliparous females

Further investigation of the additional effects of prepregnancy stress on postpartum behaviors in another cohort of BALB/cAJcl mice again replicated the findings that lactating females exhibited significantly decreased distance traveled in the open field test and increased depression-related behavior in the forced swim test compared with nulliparous females (Fig. 8; for detailed statistical results, see Additional file 1: Table S4). Additionally, chronic restraint stress experience caused decreased distance traveled in the light box (Fig. 8f) and decreased time spent in the light box (Fig. 8g), indicating increased anxiety-like behavior in lactating females that experienced prepregnancy stress.

### Body weight and food consumption during the peripartum period

For body weights during the postpartum period in the four strains (Additional file 4: Figure S3A), there were significant main effects of strain (*F*_3,142_ = 46.65, *p* < 0.0001) and reproductive state (*F*_2,142_ = 206.16, *p* < 0.0001) and significant interactions of strain × day (*F*_6,284_ = 2.58, *p* = 0.0191), reproductive state × day (*F*_4,284_ = 116.87, *p* < 0.0001), and strain × reproductive state × day (*F*_12,284_ = 1.87, *p* = 0.0379). Post hoc analyses showed that, generally, in each reproductive state group, the two BALB/cA substrains had increased body weights compared with the two C57BL/6 substrains. In addition, the body weights of the nonlactating groups in each strain decreased after pup removal on PD1, and the body weights of nonlactating mice were significantly greater than those of nulliparous females in the B6 substrains, although there were no significant differences after Bonferroni correction between the two groups in the BALB/c substrains on PD3 and PD8. Lactating females showed increased body weights compared with nonlactating and nulliparous females in each strain. Regarding food consumption (Additional file 4: Figure S3B), there were significant main effects of strain (*F*_3,142_ = 6.33, *p* = 0.0005) and reproductive state (*F*_2,142_ = 695.78, *p* < 0.0001) and significant interactions of reproductive state × day (*F*_2,142_ = 172.10, *p* < 0.0001) and strain × reproductive state × day (*F*_6,142_ = 2.75, *p* = 0.0146). Post hoc analyses revealed that lactating females showed significantly increased food consumption compared with nulliparous and nonlactating females in each strain. In nulliparous and nonlactating females, there were no significant strain differences in food consumption. For lactating females, food consumption increased across days. Lactating CAJcl females exhibited increased food consumption compared with the other three strains of lactating females on PD1–3, although the difference between the B6JJcl mice and the CAJcl mice did not reach a significance after Bonferroni correction. Lactating B6JJcl and CAJcl females showed significantly increased food consumption compared with lactating CACrlj females on PD8–10.

In another cohort of BALB/cAJcl females with or without stress experience, there were significant main effects of reproductive state (*F*_1,46_ = 185.83, *p* < 0.0001) and stress (*F*_1,46_ = 21.86, *p* < 0.0001), significant interactions of reproductive state × day (*F*_8,368_ = 419.03, *p* < 0.0001), stress × day (*F*_8,368_ = 18.01, *p* < 0.0001), and reproductive state × stress × day (*F*_8,368_ = 5.79, *p* < 0.0001) on body weights (Additional file 4: Figure S3C). Post hoc analyses revealed that CRS-nulliparous females showed decreased body weights one week after the start of the stress session compared with NS-nulliparous females, whereas no significant differences in body weights were found between the two groups after the stress session. In lactating females, CRS-induced decreases in body weights were found after the peripartum period, but a significant difference was not observed on PD14. For food consumption (Additional file 4: Figure S3D), there was a significant main effect of reproductive state (*F*_1,46_ = 477.89, *p* < 0.0001) and a significant interaction of reproductive state × day (*F*_4,184_ = 199.22, *p* < 0.0001). NS- and CRS-lactating females consumed more food during the prepartum and postpartum periods, except for at PD1, than NS- and CRS-nulliparous females.

## Discussion

This study examined pregnancy- and lactation-induced changes in anxiety-like and depression-related behaviors in four inbred strains (C57BL/6J, C57BL/6JJcl, BALB/cAnNCrlCrlj, and BALB/cAJcl) of female mice to explore a potential animal model of postpartum depression. The overall findings of this study were that there are strain and substrain differences among the inbred strains in all the behaviors examined and that there are reproductive state-related differences in those behaviors, depending on the strain and presence of pups. In general, females of the two BALB/c substrains showed decreased locomotor activity, increased anxiety-like behavior, and increased depression-related behavior compared with females of the two C57BL/6 substrains. Compared with CAJcl females, CACrlj females exhibited decreased locomotor activity, slightly decreased anxiety-like behavior, and increased depression-related behaviors. C57BL/6J females showed lower locomotor activity and more anxiety-like behavior than C57BL/6JJcl females. Furthermore, decreased locomotor activity and/or increased depression-related behavior were found in the lactating females compared with females in other reproductive states in some inbred strains, especially in BALB/cAJcl strain.

Inbred strains have been previously compared to determine genetic influences on behavior in rodents [55–57]. It is assumed that strain differences in behavior may be accounted for by genetic differences between inbred strains. In the open field test, C57BL/6 females traveled longer distances and spent more time in the center area than BALB/c females. The time spent in the center has been used as a measure of emotionality/anxiety in novel and open environments [47]. The longer time spent in the center of the open field by C57BL/6 females than by BALB/c females might reflect the lower level of anxiety-like behavior. Similar strain differences were observed in the other two tests, namely, the light/dark transition test and the elevated plus maze test, which have also been commonly used for assessing anxiety-like behavior in mice [28, 58, 59]. C57BL/6 females showed more locomotor activity and less anxiety-like behavior than BALB/c females, as indicated by the longer distances traveled in the light and dark chambers and the greater number of transitions between chambers in the light/dark transition test and by the longer distances traveled, the greater number of entries into open arms, and the longer time spent in open arms in the elevated plus maze test. These results are largely consistent with previous findings in males [25, 60–63] and females [63, 64], supporting the view that C57BL/6 mice are more active and “less emotional/anxious”, whereas BALB/c mice are less active and “more emotional/anxious” to novel environments [63, 65, 66]. However, in our study, a longer time spent in the light box in the light/dark transition test, which is also considered a measure of anxiety-like behavior [67], was also observed in BALB/c females compared with in C57BL/6 females, suggesting a lower anxiety-like phenotype in BALB/c females. Given the results of other behavioral measures in our study and the previous reports described above, further examination and interpretation of the behavioral traits of BALB/c females in the light/dark transition test are warranted.

Strain differences were found in the behavioral tests for assessing depression-related behavior. In the Porsolt forced swim test, CACrlj females exhibited greater immobility and traveled shorter distances than C57BL/6 females. In contrast, CAJcl females exhibited less immobility than C57BL/6 females, although CAJcl females traveled shorter distances than C57BL/6 females. In addition, BALB/c females showed lower sucrose preference than C57BL/6 females. These results suggest that BALB/c females exhibited increased depression-related behavior and anhedonia-like behavior compared with C57BL/6 females. Such differences in immobility in the forced swim test between male C57BL/6 and BALB/c mice have been reported, although previous studies have been inconsistent, as increased immobility time in BALB/c mice [62, 68], decreased immobility time in BALB/c mice [69, 70], and no statistically significant differences between the two strains [71, 72] have all been reported. Although the precise reasons for these inconsistent findings are unclear, differences in genetic background, housing conditions, and testing conditions might contribute to the variation in behaviors of inbred strains of mice.

There are many substrains of C57BL/6 and BALB/c that have been developed and maintained by various investigators and vendors [73, 74]. The major substrains of C57BL/6 are C57BL/6J, which is known as the original JAX mouse strain, and C57BL/6N, which was separated from the C57BL/6J strain. Such substrains show genetic differences [75, 76] and variations in various types of behaviors [77, 78]. The C57BL/6J mice used in this study, which were obtained from Charles River Laboratories Japan, an authorized distributor and breeder of JAX mice strains, had a pure C57BL/6J background because they had been bred and maintained according to JAX’s genetic stability program [79]. The mice of the C57BL/6JJcl strain, supplied by CLEA Japan, were derived from progeny of C57BL/6J mice that were introduced to CLEA Japan. Genetic differences between C57BL/6J and C57BL/6JJcl have been reported [75]. Thus, substrain-related differences in behavior between the two C57BL/6 substrains and between the two BALB/c substrains could be explained by genetic variations between the substrains. However, we cannot exclude the possibility that there were possible influences of different environmental conditions on the behavioral differences because the substrains of mice were bred and housed in different animal facilities by the respective vendors until arrival at our laboratory.

This study reveals that there are reproductive state-dependent differences in locomotor activity, anxiety-like behavior, and depression-related behavior in some inbred strains of mice. In the B6JJcl strain, nonlactating primiparous females showed a tendency to display decreased locomotor activity in the open field test when compared with nulliparous females. In B6J and CAJcl strains, nonlactating primiparous females showed more immobility time and/or shorter distances traveled than nulliparous females. These results suggest that reproductive experience, such as pregnancy and parturition, could result in reduced locomotor activity and increased depression-related behavior in female mice in some inbred strains. These behavioral changes might be due to possible changes in the brain or endocrine system underlying the reproductive process but cannot be explained by interactions with pups because the nonlactating females were deprived of their pups one day after parturition. Similarly, in those strains, decreased locomotor activity and increased depression-related behavior were found in lactating females compared with nulliparous females. In the CAJcl strain, lactating females showed decreased distance traveled in novel environments and increased immobility in the forced swim test. Increased immobility in the forced swim test in lactating females was also observed in the B6J strain. In the B6JJcl and CAJcl strains, lactating females showed a reduced sucrose preference or anhedonia-like behavior compared with their nulliparous female counterparts. In the forced swim test, nonlactating and lactating primiparous females showed greater immobility and shorter distance traveled compared with nulliparous females in CAJcl and B6J strains. Interestingly, nonlactating primiparous females did not differ from nulliparous females in CAJcl strain in terms of distance traveled in the open field test, suggesting similar general locomotor activity. Moreover, there were no differences in distance traveled among B6J females in the different reproductive states. These results suggest that general locomotor activity might not affect the observed increase in immobility. It is a well-known problem that conducting behavioral tests, even with identical apparatus and test protocols using same inbred mice, may not guarantee identical results; unknown environmental factors could contribute to variations in behavioral outcomes [80, 81]. Although sources of the environmental factors are unclear, it is crucial to show which results are highly statistically significant, robust, and reliable. Thus, we reevaluated behavioral phenotypes in independent cohorts of mice to confirm the reliability of our results. The results showed that some statistically significant differences between nulliparous and lactating primiparous females in the first cohort of CAJcl mice were not observed in another independent cohort of mice. For example, the marginally significant decrease in the sucrose preference found in lactating females on the first examination of the four inbred strains was not observed in the other experiments. One possibility is that the estrous cycle that could cause variations in behaviors, especially in nulliparous females ([64, 82, 83]; but [64, 84, 85]), was not controlled in this study. Nevertheless, the results that the reduced locomotor activity and the increased depression-related behavior in lactating CAJcl females were replicated in two independent experiments in this study and in our unpublished data, which indicate the robustness and reliability of the results. In the additional experiment, chronic stress prior to pregnancy further induced increased anxiety-like behavior in the light/dark transition test in lactating CAJcl females, which is consistent with results regarding the postpartum behaviors of BALB/cJ females previously exposed to chronic stress in the novelty-suppressed feeding test [24], which is a test used for assessing anxiety-like behavior [86]. In contrast, prior stress experience had no effect on the behaviors of nulliparous females. Previous studies in male rats and mice have reported that chronic stress-induced behavioral changes returned to normal within 3 weeks [87–90]. The lack of effects of chronic stress in nulliparous females observed in this study might be owing to testing their behaviors more than 3 weeks after the last session of chronic stress procedure in this study. These findings further highlight the impact of the combined effects of prepregnancy stress and reproductive experience on the development of the depressive state in lactating BALB/cAJcl female mice.

Interaction with pups plays an important role in the onset and maintenance of maternal behavior in female rats [91–94]. When virgin female rats are exposed to unfamiliar pups, they initially tend to avoid the pups or show approach/withdrawal responses to them [91, 92]. However, continuous exposure to pups facilitates maternal responsiveness to pups in female rats [91]. The transition from avoidance of pups to pup-directed behavior is suggested to be associated with reduced levels of anxiety and fear [3, 39]. In this line of research, it has been reported that lactating rats exhibit less anxiety-like behavior than virgin females in different types of behavioral tests [39–42], and recent physical interactions with pups are necessary for reduced anxiety-like behavior [44]. In contrast, it has also been reported that in the hole-board test, another type of test for assessing anxiety-like behavior, lactating female rats selectively bred for high and low anxiety-related behavior in the elevated plus maze showed higher anxiety-like behavior than virgin females [38]. In outbred Swiss mice, lactating females exhibited less anxiety-like behavior than virgin females in the light/dark transition test [43]. In contrast, our results of the three different tests for assessing anxiety-like behavior indicated that there were no obvious differences in anxiety-like behavior among reproductive states in the C57BL/6 and BALB/c strains. However, decreased locomotor activity to a novel environment and avoidance of the center open space, which are suggestive of increased anxiety-like behavior, were observed in lactating CAJcl females. Lactating females also exhibited increased vertical activity in the open field test compared with nulliparous females. Some investigators have viewed vertical activity or rearing as indices of escape behavior and behavioral excitability [95]. These findings suggest that the emotional responses to novel environments vary with reproductive state and are dependent on genetic background and the testing situation. Moreover, our data regarding the comparison of nonlactating females with lactating females suggest that interactions with pups can induce increased depression/anhedonia-related behaviors in B6JJcl and CAJcl primiparous females and decreased locomotor activity in CAJcl primiparous females. In lactating females, body weight and food consumption increased during lactation in each strain, whereas pup removal induced decreases in body weight and food consumption in nonlactating females. During lactation, energy storage and energy intake are needed to produce milk and to care for pups, which might result in fatigue or loss of energy, which are possible factors associated with a depressive state.

Our understanding of the neurobiological mechanisms underlying PPD is still limited because of the paucity of valid animal models for PPD and lack of research using these animal models, as recently reviewed by Brummelte and Galea [1, 96] and Pawluski et al. [8]. During the peripartum period, fluctuations in gonadal hormones (estradiol and progesterone), changes in oxytocin levels, and dysregulation of hypothalamus-pituitary-adrenal axis function (such as chronic glucocorticoid hypersecretion) due to hormonal fluctuations and stress are hypothesized to be associated with the development of PPD (see [1, 8, 96]). Using animal models that mimic the hormonal changes and stress effects of PPD, some studies have reported suppressed adult neurogenesis [97], morphological changes in dendritic spines [98], and altered expression of genes including BDNF, serotonin transporter, and GABAA receptors [18] in the hippocampus that is one of the brain regions associated with PPD [8]. Our current study provides potential mouse models to examine the underlying neurobiological mechanisms of PPD.

The results of this study revealed that there are strain differences in locomotor activity, anxiety-like behavior, and depression-related behavior among the four tested inbred strains (C57BL/6J, C57BL/6JJcl, BALB/cAnNCrlCrlj, and BALB/cAJcl) of female mice, suggesting a genetic contribution to the regulation of these behaviors in female mice. To our knowledge, our data are the first to indicate that there are differences in some of these behaviors among the substrains used in this study. These findings indicate the importance of the influences of genetic variations and of possible genetic drift on the behavioral characteristics of each inbred strain. Furthermore, this study demonstrated that there were pregnancy- and lactation-induced changes in different behavioral domains in some inbred strains. The marked behavioral differences between nulliparous and lactating primiparous females were observed in the BALB/cAJcl strain, in which lactating females exhibited decreased locomotor activity and increased depression-related behavior. We confirmed that other independent experiments yielded consistent and replicable results for the behavioral differences between reproductive states in the BALB/cAJcl strain. Together, these findings suggest that lactating females of some inbred strains of mice are potential animal models of a genetic predisposition to the depression-related states during the postpartum period and, thus, may be useful models for further investigations of the genetic and neurobiological mechanisms underlying the development of postpartum depression.

## Additional files

Additional file 1: **Table S1.** Statistical analyses of behavioral data in females of four inbred strains of mice (C57BL/6J, C57BL/6JJcl, BALB/cAnNCrlCrlj, and BALB/cAJcl). **Table S2.** Number of mice showing tail climbing behavior for more than 30 s in the tail suspension test. **Table S3.** Statistical analyses of behavioral data of nulliparous and lactating primiparous BALB/cAJcl female mice. **Table S4.** Statistical analyses of behavioral data in BALB/cAJcl female mice previously exposed to chronic restraint stress. (XLSX 30 kb)

Additional file 2: **Figure S1.** Depression-related behavior in BALB/c and C57BL/6 female mice during the postpartum period in the Porsolt forced swim test. The Porsolt forced swim test was performed for a 10-min test period in nulliparous, nonlactating primiparous, and lactating primiparous females of C57BL/6J, C57BL/6JJcl, BALB/cAnNCrlCrlj, BALB/cAJcl strains. (A–D) Porsolt forced swim test: (A) immobility time (%) on days 1 and 2 and (B) distance traveled (cm) on days 1 and 2 for each 1-min block of the test session. Values are means ± SEM. (EPS 723 kb)

Additional file 3: **Figure S2.** Depression-related behavior in BALB/c and C57BL/6 female mice during the postpartum period in the tail suspension test. The tail suspension test was performed for a 10-min test period in nulliparous, nonlactating primiparous, and lactating primiparous females of C57BL/6J, C57BL/6JJcl, BALB/cAnNCrlCrlj, BALB/cAJcl strains. Time of tail-climbing behavior (%) was measured during the 10-min test period in the tail suspension test (A). Immobility time (%) was calculated in females in the CACrlj strain (nulliparous females, *n* = 16; nonlactating females, *n* = 12; lactating females, *n* = 15) and the CAJcl strain (nulliparous females, *n* = 7; nonlactating females, *n* = 9; lactating females, *n* = 13) after excluding mice showing tail-climbing behavior for more than 30 s in total during the test period. Values are means ± SEM. (EPS 250 kb)

Additional file 4: **Figure S3.** Body weight and food consumption in BALB/c and C57BL/6 female mice. Body weight and food consumption were measured in nulliparous, nonlactating primiparous, and lactating primiparous females of C57BL/6 J, C57BL/6 JJcl, BALB/cAnNCrlCrlj, BALB/cAJcl strains. (A) Body weight (g) and (B) food consumption (g) in the three reproductive groups in the four strains during the postpartum period. In another cohort of BALB/cAJcl females previously exposed to chronic stress, their body weights and food consumption were monitored during the stress period and/or during the peripartum period. (C) Body weight (g) and (D) food consumption (g) in the nonstressed (NS) nulliparous, chronic restraint-stressed (CRS) nulliparous, NS-lactating, and CRS-lactating females. Values are means ± SEM. (A,B) in each strain, *p < 0.05 after Bonferroni correction ( lactating primiparous vs. nulliparous); †*p* < 0.05 after Bonferroni correction (lactating primiparous vs. nonlactating primiparous); §*p* < 0.05 after Bonferroni correction (nonlactating primiparous vs. nulliparous). (C) *p < 0.05, comparison between nonstressed lactating and stressed lactating groups; †*p* < 0.05, comparison between nonstressed nulliparous and stressed primiparous groups. (EPS 388 kb)

## Data Availability

The datasets generated during the current study are available in the Mouse Phenotype Database (http://www.mouse-phenotype.org/). The datasets used during the current study are available from the corresponding author on reasonable request.

## Abbreviations

B6J: C57BL/6J
B6JJcl: C57BL/6JJcl
CACrlj: BALB/cAnNCrlCrlj
CAJcl: BALB/cAJcl
CRS: Chronic restraint stress
PPD: Postpartum depression
